# Optimization of household medical waste recycling logistics routes: Considering contamination risks

**DOI:** 10.1371/journal.pone.0311582

**Published:** 2024-10-07

**Authors:** Jihui Hu, Ying Zhang, Yanqiu Liu, Jiaqi Hou, Aobei Zhang

**Affiliations:** School of Management, Shenyang University of Technology, Shenyang, Liaoning, China; University of Vienna: Universitat Wien, AUSTRIA

## Abstract

The escalating generation of household medical waste, a byproduct of industrialization and global population growth, has rendered its transportation and logistics management a critical societal concern. This study delves into the optimization of routes for vehicles within the household medical waste logistics network, a response to the imperative of managing this waste effectively. The potential for environmental and public health hazards due to improper waste disposal is acknowledged, prompting the incorporation of contamination risk, influenced by transport duration, waste volume, and wind velocity, into the analysis. To enhance the realism of the simulation, traffic congestion is integrated into the vehicle speed function, reflecting the urban roads’ variability. Subsequently, a Bi-objective mixed-integer programming model is formulated to concurrently minimize total operational costs and environmental pollution risks. The complexity inherent in the optimization problem has motivated the development of the Adaptive Hybrid Artificial Fish Swarming Algorithm with Non-Dominated Sorting (AH-NSAFSA). This algorithm employs a sophisticated approach, amalgamating congestion distance and individual ranking to discern optimal solutions from the population. It incorporates a decay function to facilitate an adaptive iterative process, enhancing the algorithm’s convergence properties. Furthermore, it leverages the concept of crossover-induced elimination to preserve the genetic diversity and overall robustness of the solution set. The empirical evaluation of AH-NSAFSA is conducted using a test set derived from the Solomon dataset, demonstrating the algorithm’s capability to generate feasible non-dominated solutions for household medical waste recycling path planning. Comparative analysis with the Non-dominated Sorted Artificial Fish Swarm Algorithm (NSAFSA) and Non-dominated Sorted Genetic Algorithm II (NSGA-II) across metrics such as MID, SM, NOS, and CT reveals that AH-NSAFSA excels in MID, SM, and NOS, and surpasses NSAFSA in CT, albeit slightly underperforming relative to NSGA-II. The study’s holistic approach to waste recycling route planning, which integrates cost-effectiveness with pollution risk and traffic congestion considerations, offers substantial support for enterprises in formulating sustainable green development strategies. AH-NSAFSA offers an eco-efficient, holistic approach to medical waste recycling, advancing sustainable management practices.

## 1. Introduction

Under the combined pressure of industrialization and population growth, global waste generation is increasing alarmingly [1]. Medical waste, in particular, has become an urgent environmental problem due to its potential environmental and health risks. The widespread use of disposable medical devices by the healthcare industry, a non-negligible part of the waste stream, further exacerbates the challenge of environmental sustainability. Although the healthcare sector is increasingly adopting environmentally friendly materials, it still faces difficulties in achieving sustainable recycling and reuse.

**Fig 1:**
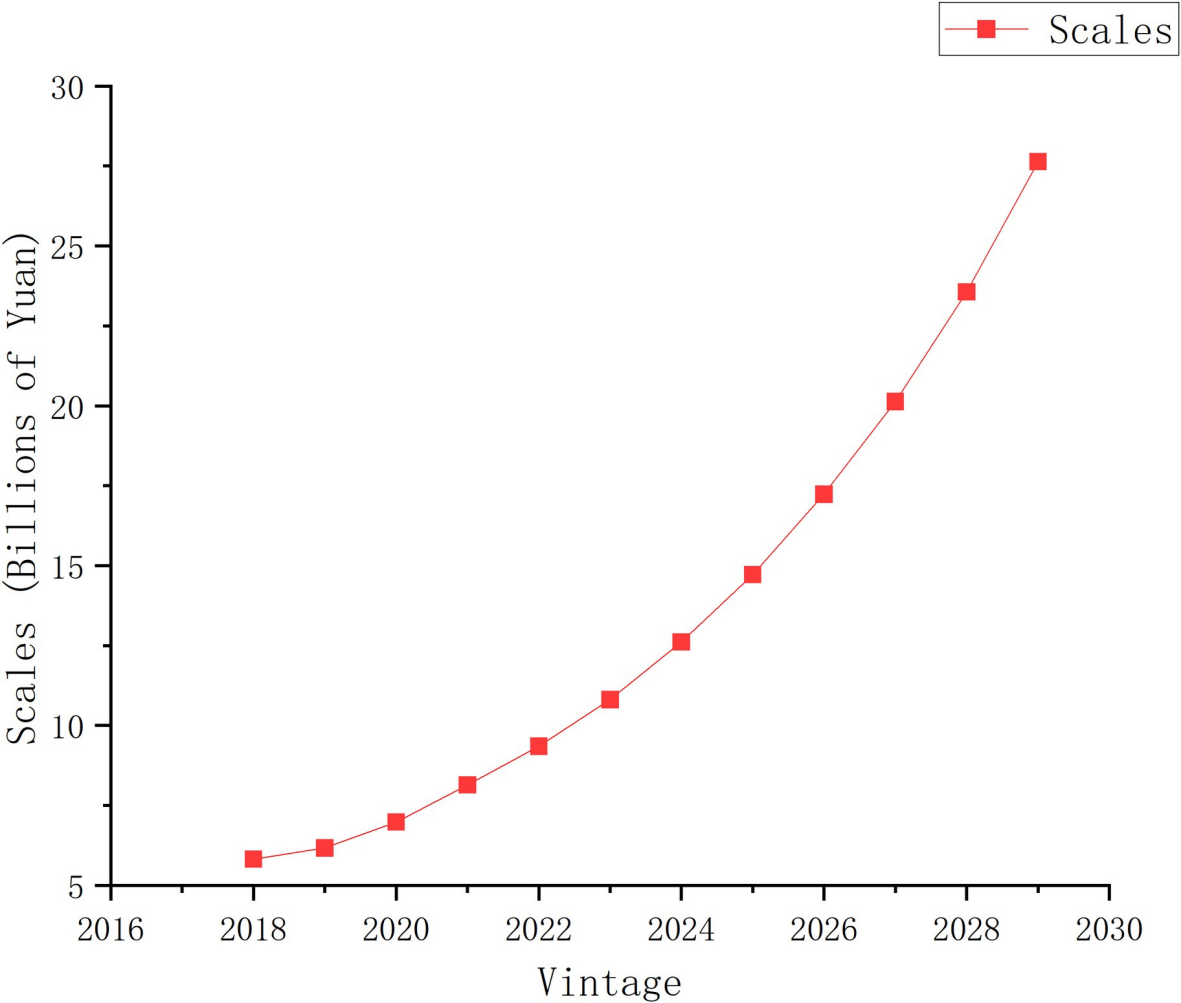
China medical waste disposal market size statistics and forecast, 2018 to 2029.

The expansion of the medical waste disposal market, especially in China, is evident from the analysis of market size statistics and forecasts from 2018 to 2029, as shown in Fig 1 [2,3]. These figures not only reveal the growth in market size but also reflect the urgent need for effective management solutions. With changing demographics, especially aging and increasing demand for home care services, the generation of household medical waste has risen significantly, which poses a serious threat to public health, safety, and the environment. It has therefore become essential to put in place stringent measures for the segregation and management of household medical waste, especially in dealing with sharps and accidental blood exposure.

Household medical waste is usually classified into four main categories: chemical, pharmaceutical, hazardous, and infectious waste, of which about 10 to 25 percent are biotoxic, corrosive, infectious, or unsafe. Improper disposal may lead to environmental pollution and disease transmission, posing a threat to human health [4,5]. For example, during the COVID-19 pandemic, used protective gear that was improperly discarded could have led to socio-environmental problems due to contamination and disease transmission [6]. It is worth emphasizing that when hazardous household medical waste is mixed with other household waste, the entire waste becomes hazardous. For example, contaminated protective gear such as masks may transmit diseases to staff and waste collectors when they are usually discarded with household waste. Household medical waste recycling is therefore an important waste collection and management service.

Due to the rapid increase in the volume of household medical waste, systematic management of the waste is problematic. Globally, many countries have problems in the management of household medical waste, and these problems add to the complexity and urgency of management [7–12]. These problems include illegal secondary sales, arbitrary disposal, mixing with household waste, and may even involve black industry chains [13]. To address these challenges, the World Health Organization (WTO) has provided an exhaustive reference manual on medical waste management, details of which are available at https://www.who.int/news-room/fact-sheets/detail/health-care-waste. This manual covers many aspects of the proper disposal of medical waste and emphasizes global co-responsibility. Therefore, the safe disposal and effective management of household medical waste is not only an important issue of environmental protection and public health but also a key to safeguarding human health and ecological security. This study aims to examine in depth the current situation, challenges, and possible improvement strategies in the management of household medical waste, to provide a scientific basis for formulating more effective management measures.

The main contribution of this paper is to improve the household medical waste recycling process through a scientifically rigorous vehicle path optimization method. Enhancing vehicle route planning provides strong support to establish a safe, efficient, and ecologically sound medical waste management system. Therefore, based on the characteristics of household medical waste, this paper develops a vehicle path problem for household medical waste recycling. The problem takes into account the hazards that may be caused to the local community and the environment during the transport of household medical waste recycling, especially considering that the number of pollutants spilled varies with the wind speed and the amount of waste. To more closely match the actual urban traffic conditions, a road congestion factor was introduced and parameterized in the study to simulate the actual traffic congestion. By analyzing the distribution and collection points of household medical waste, the collection routes of vehicles are optimized to reduce unnecessary traveling distances, thus reducing the total expenditure while mitigating the impact of environmental pollution. This also responds to the pragmatic requirement of balancing fiscal efficiency and ecological protection.

Furthermore, in this paper, an Adaptive Hybrid Artificial Fish Swarm Algorithm with Non-Dominated Sorting (AH-NSAFSA) is developed to solve the proposed model. The algorithm evaluates and ranks the solution set using a non-dominated ranking technique to determine the most appropriate solution. To improve the performance of the algorithm, several innovations are introduced in this study, including a decay function for the three parameters of the artificial fish field of view, the step size, and the crowding factor, which works together to improve the convergence speed and search accuracy of the algorithm. In addition, an elimination behavior was introduced through the crossover operation to ensure the diversity of the population and the quality of the solution. To ensure the effectiveness of the proposed model and algorithm, 31 nodes were selected from the RC101 category of the Solomon dataset for testing. The experimental results of the algorithm were evaluated and discussed through a comprehensive series of experiments that not only validated the effectiveness of the model and algorithm but also demonstrated the potential of the algorithm in solving practical problems. In conclusion, by proposing and validating a bi-objective optimization model, this study provides an innovative solution to the household medical waste recycling pathway problem, which helps to reduce operational costs and mitigate environmental pollution, thus promoting sustainable development.

The following is an arrangement of the study’s contents to provide a theoretical framework for the following investigation. Section 2 will thoroughly classify the pertinent medical waste recycling reverse logistics literature. In Section 3, the research challenge of this work will be elucidated in greater detail, and a mathematical model of home medical waste recycling will be constructed to perform a quantitative assessment of the significant components of the recycling process. Based on the accepted mathematical model and the conventional artificial fish swarm algorithm, Section 4 will In Section 4, an adaptive hybrid artificial fish swarm algorithm with non-dominated sorting is presented to increase recycling efficiency and optimize the recycling path. In Section 5, numerical experiments will be designed to verify the viability and efficacy of the proposed model and algorithm. In Section 6, the key findings of this study will be summarized, and future research directions will be considered to provide a more effective and scientific solution for the recycling management of household medical waste.

## 2. Literature review

### 2.1 Related studies

The current increase in medical waste generation poses challenges for cities regarding the management of collection, treatment, recycling, and disposal. Collection, transportation, treatment, and recycling are significant medical waste management costs. The medical waste collection problem is a variant of the Vehicle Route Problem (VRP). If medical waste is not disposed of properly, it may have adverse effects on the environment and human health [14]. Medical waste management is a reverse logistics management problem designed for medical waste. When medical waste logistics is problematic, there will be persistent environmental and human health risks [15], such as respiratory diseases, carcinogenicity, and other problems [16]. In response to the inherent risks entailed in the transportation of medical waste, academicians have explored and examined the reverse logistics pertaining to the collection of such waste. For example, SHI et al. discussed the importance of medical waste management. They proposed a new mixed-integer linear programming model to improve the reverse logistics network design for medical waste management, aiming to effectively manage medical waste and reduce its environmental and public health risks [17]. Wang et al. addressed the problem of reverse logistics network design for municipal medical waste by combining the grey prediction model GM (1,1) and a MO-optimization model. They proposed a dynamic approach to make facility allocation decisions for a medical waste reverse logistics network [18]. Alizadeh et al. considered forward and reverse logistics networks, emphasizing medical waste sorting and recycling while considering biological risks [19]. Nikzamir et al. proposed a new medical waste siting pathway problem, considering the stochastic nature of pollution emission during the transportation of medical waste from medical centers to treatment centers, and proposed a Multi-Objective Water Flow Algorithm (MOWFA) based on the analytical hierarchy process (AHP) to solve the problem [20]. Kargar et al. considered the uncertainty in the amount of medical waste generated and used a fuzzy goal-planning approach to deal with the uncertain parameters in the model [21]. Torkayesh et al. proposed a MO-optimization model considering economic, environmental, and social aspects to facilitate the application of sustainability objectives in healthcare waste management systems [22]. Govindan et al. applied queuing theory for the first time to trucks managing waiting times at processing centers, considering both vehicle paths and center locations, and used a scenario-based approach to deal with uncertainty in waste generation [23]. Kargar et al. proposed a tri-objective function model and validated the model with a real-life case study in Iran [24]. Singh et al. proposed a two-stage hybrid decision-making framework for selecting third-party reverse logistics providers and optimizing order allocation in the post-epidemic era [25]. Wang et al. proposed a BVM-based risk assessment model for identifying and controlling key risk factors in the reverse logistics of medical waste [26]. Wang et al. proposed a medical waste collection path optimization model considering infection risk and multiple disposal centers, aiming to minimize the maximum infection risk and transportation cost [27]. Nasreddine proposed a new medical waste transport model based on the Multi-Compartment Vehicle Routing Problem (MC-VRP), which separates hazardous waste from non-hazardous waste along the transport route [28]. Lin et al. proposed an adaptive MO algorithm for the problem of the path of an electric vehicle in the management of medical waste [29]. Hajer et al. studied the task of transporting healthcare waste (HCW) and presented, for the first time, a route planning problem based on multi-chamber vehicles to minimize the total distance traveled in the context of HCW transport [30]. Qi et al. developed a three-tiered recycling network model to investigate the establishment of a professional medical waste reverse logistics network [31]. Considering routine and public health emergencies, Zhu et al. investigated optimizing a reverse logistics network for healthcare waste under uncertain supply-demand conditions [32]. A route optimization problem for medical waste collection with temporary storage risk and sequential uncertain service requests was introduced by Zhang et al. [33]. Zhang et al. proposed a time window model for the multi-cycle medical waste recycling vehicle path problem for the municipal medical waste problem. They designed an improved neighborhood search algorithm to improve efficiency and reduce the carbon footprint [34].

Many scientists have adopted new approaches to address the issue of medical waste management. Zhao et al. applied data mining techniques and intelligent algorithms to construct a multi-period emergency disposal logistics network optimization under uncertainty conditions model, taking into account the uncertainties in the amount of medical waste generated and the region’s population density [35]. Keyvan et al. developed a planimetric clustering routing method to optimize healthcare waste collection using a Geospatial Information System (GIS) [36]. Zineb et al. proposed a novel healthcare waste management system based on collaboration and technological advances, using XAI technology and vehicle optimization algorithms to improve waste management efficiency [37]. Kaviya et al. propose a sustainable, green, circular economy model for controlling waste and emissions in healthcare systems using AI technology and drone technology [38]. Some scholars have established a reverse logistics optimization model with local characteristics by considering various local factors. Anna et al. discussed the importance of logistics constraints in medical waste management systems. Using Poland as an example, they analyzed the impact of legal requirements, organizational factors, and economic aspects [39]. Budak et al. proposed a new reverse logistics optimization model for the specific case of Turkey and examined the impact of waste volume changes on the optimal reverse logistics network through sensitivity analysis [40]. He et al. discussed the logistics of medical waste collection in China and proposed a design and optimization method to improve the medical waste collection network [41]. Pereira et al. discussed the phenomenon of household waste medicines (HWM), its impact on the environment and public health, and the reverse logistics system in Brazil [42]. Balci et al. designed a multi-purpose reverse logistics network to manage healthcare waste generated in Istanbul [43]. Dhingra et al., for the first time, identified and analyzed the challenges of adopting blockchain technology in healthcare waste management by combining the BWM and DEMATEL methodologies in the context of healthcare in India [44]. Trivedi et al. proposed an optimization model based on objective planning to manage recycling operations for medical waste generated during mass immunization campaigns [45].

On the other hand, scholars have addressed the problem of medical waste collection during pandemic outbreaks. Yu et al. innovatively proposed a model for managing medical waste during the COVID-19 outbreak and demonstrated the application of the model through a case study [46]. Tirkolaee et al. addressed the pandemic healthcare waste collection and transportation problem during the pandemic and proposed a sustainable model considering cost-effectiveness and environmental impacts [47]. Luo et al. investigated the problem of reverse logistics network design for infectious medical waste (IMW) during the COVID-19 outbreak and proposed a collaborative multi-participant (public center hospitals, disposal facilities, logistics providers, and government) location and path optimization model [48]. Erdem et al. designed a sustainable logistics network for collecting and transporting hazardous medical waste generated during the COVID-19 pandemic, introducing an electric medical waste collection vehicle path problem to optimize the routes and shifts of the electric vehicles while considering the choice of charging technology [49]. Liu et al. combined a SEIRD dynamic model and particle swarm optimization algorithm to propose a flexible emergency logistics network model to adapt to real-time changes in the epidemic region [50]. Karatas et al. explored transportation and location planning problems that arise during epidemics and their solutions [51]. Yaspal et al. introduced a data-driven approach to digital transformation. They used a MO-optimization model to manage healthcare waste, focusing on strategies to reduce the accumulation of healthcare waste [52]. Nosrati et al. introduced the concept of epidemic disruption to determine the amount of waste generated in network facilities. They formulated a bi-objective mixed integer linear programming model for designing a reverse logistics network for healthcare waste management under uncertainty and epidemic disruption [53]. Naeme et al. considered the uncertainty in waste generation rates associated with COVID-19, using a plausibility-based likelihood planning approach to deal with the uncertainty [54]. Elham et al. proposed a complex integer linear programming model based on the Cuckoo optimization algorithm to design a reverse logistics network for COVID-19 waste management [55]. Cao et al. proposed a digital twin-driven, robust two-layer optimization model for the COVID-19 medical waste location-transport problem [56]. A hybrid two-step approach combining infectious disease modeling and multi-criteria decision-making for predicting and transporting infectious medical waste during the COVID-19 pandemic was presented by Li et al. [57]. For the management of medical waste during the COVID-19 outbreak, Kannan et al. developed a two-objective mixed-integer linear programming model [58]. Hamed et al. COVID-19 medical waste management is optimized using objective and robust possibility planning [59]. Eren et al. investigated the problem of safe distance-based vehicle routing for medical waste collection during the COVID-19 pandemic, combining safety scores and total transport distances [60]. Cao et al. proposed a sustainability-oriented integrated location-transport optimization problem for multi-phase, multi-type disaster medical waste during the COVID-19 pandemic [61]. Mei et al. developed a multi-period medical waste emergency reverse logistics network siting model to account for the characteristics of medical waste volume growth during the COVID-19 epidemic [62]. Cao et al. investigated how to optimize a two-stage medical waste transport network during the COVID-19 outbreak, considering factors such as the choice of multiple vehicles, sustainability, and the probability of infection [63].

In solving the problem of reverse logistics of medical waste, scientists have used various approaches. Alireca et al. developed three new multi-objective optimization algorithms for solving the non-permutation flow-shop scheduling problems: the Multi-objective Ant Lion optimization algorithm (MOALO), the Multi-objective Keshtel Algorithm (MOKA) and Multi-objective Keshtel and Social Engineering Optimizer (MOKSEA), and experimentally adjusted the algorithm parameters to optimize the model [64]. Alireca et al. implemented the Accelerated Benders decomposition algorithm to address the complexity of the model for a portfolio-based closed-loop supply chain network for Dairy products and used the weighted sum method (WSM), augmented ε-constraint (AEC), and fuzzy Mult objective programming (FMOP) to deal with the bi-objectivity of the model [65]. In addition to the Multi-Objective Water Flow Algorithm (MOWFA) [20], Best-Worst Method (BWM) [26], Multi-Attribute Decision-Making Approach (MADMA) [25], Enhanced Epsilon Constraints Approach [48], Adaptive Large Neighborhood Search (ALNS) [49], Particle Swarm Optimization (PSO) [50], Cuckoo Optimization Algorithm (COA) [55], etc.

### 2.2 Research gap analysis and contributions

In summary, the existing literature provides a solid foundation for an in-depth study of household medical waste transport logistics. S1 Table. demonstrates some of the previous research results. Through a meticulous literature review, this paper draws the following conclusions:

1. the inadequacy of pollution risk research: In the field of medical waste transport logistics, insufficient attention has been paid to the risk of pollution in the transport process, and most of the existing studies simplify the pollution risk into a single-factor function for estimation, failing to capture the multidimensional characteristics of the risk comprehensively.
2. Neglect of Household Medical Waste Recycling: Existing studies have generally neglected the importance of household medical waste recycling, which may be related to the insufficient understanding of the characteristics of household source waste and its potential impact on the environment and public health.
3. Translation problems in MO-optimization studies: When considering MO-medical waste transportation problems, existing studies tend to reduce MO problems to single-objective problems, showing a lack of research on true MO-optimization methods.

Based on these findings, this study identifies key research directions and challenges in household medical waste transport logistics networks, especially considering realistic constraints such as pollutant emissions, road congestion, and wind speed.

Consequently, the main contributions of this study are summarized as follows:

1. the multi-objective mixed integer planning (BOMIP) model is extended to design a vehicle routing problem for household medical waste recycling, which can simultaneously optimize the total cost and the risk of environmental pollution.
2. road congestion is considered and incorporated into the vehicle speed function to fit the differences in vehicle speeds on different roads during real urban transport.
3. the impact of environmental pollution caused during the recycling of household medical waste is considered.
4. A pollution risk function was proposed to assess the magnitude of pollution risk by the total number of people affected. The influence of the amount of leaked pollution, the number of inhabitants along the route, the transport time, and the wind speed on the assessment of the pollution risk is also taken into account.
5. For the proposed vehicle path problem of multi-objective household medical waste recycling, the Adaptive Hybrid Artificial Fish Swarm Algorithm with Non-dominated Sorting (AH-NSAFSA) is proposed. To improve the performance of the algorithm, a decay function as well as a cancellation behavior is introduced in the proposed algorithm.

## 3. Problem formulation

A Bi-objective mixed-integer programming (BOMIP) model is developed in this section, and the household medical waste recycling routing problem is introduced. The challenge of routing medical waste recycling for households is explained in Section 3.1. The parameters and decision variables that will be incorporated into the formulation are covered in Section 3.2. A vehicle speed function is proposed in Section 3.3 to mimic the state of traffic congestion in the city. To account for the effects of the recycling trucks on the surrounding community and the environment during the transportation process, Section 3.4 suggests a pollution risk function. A pollution risk function that considers the recycling trucks’ effects on the surrounding community and environment is shown in Section 3.4. In Section 3.5, a two-objective mixed-integer programming model is developed for the household medical waste recycling routing problem. When the appropriate constraints are met, the model minimizes the transportation risk and the overall cost of transportation.

### 3.1 Problem description

A collection center and sufficient vehicle fleets aiming at recycling household medical waste are required to solve the vehicle route problem and maximize the recycling of household medical waste while considering road congestion. Each residential node’s position, the collection center’s location, and the volume of medical waste produced are all known. It is crucial to remember that the collection center is where the cars must begin. Furthermore, a few presumptions were maintained, including that all the cars were of the same make and model and that driver and load had no effect on the car’s speed. Second, vehicle damage and the time spent loading and unloading medical waste should have been considered during vehicle collection. Thirdly, there is contamination in the collected home medical waste. Lastly, specific home nodes can independently transport the medical waste to the collection center. To recycle domestic medical waste, this mathematical model for vehicle path optimization seeks to reduce fixed costs, transfer costs, incentive costs, fuel costs, and pollution risks.

### 3.2 Symbols and decision variables

The collecting centers are denoted by *P* = {1}. The group of home nodes is *Q* = {1,2,…*q*}. All nodes together make up a set *N* = *P*∪*Q*. *U* = {1,2,…,*u*} is the group of cars used to collect medical waste from homes. The group of home nodes represented by the letter *M* recycles using the collection vehicles. *E* = *P*∪*M* is the group of residential nodes that use the collection locations and the recycling vehicles.

The fixed expense paid when medical waste is collected by a collection truck is denoted by *CK*. The expense associated with gathering every medical waste unit is denoted by *CT*. The price of fuel per unit is indicated by *COF*. *PR* stands for the reward that the household node receives when it transports its medical waste to the collection center. *AW*_*j*_ is the quantity of medical waste produced at home by node *j*. *q* is the number of nodes in a home. The distance *d*_*ij*_ is the difference between nodes *i* and *j*. *u* represents the number of collection cars. The maximum load and range of the vehicles collected are indicated by letters *D* and *L*, respectively. *v* is the collection vehicles’ typical driving speed. The time it takes to get from node *i* to node *j* is indicated by *t*_*ij*_. *m* is the total number of vehicles employed for collecting.

The load of the collection vehicle *k* between node *i* and node *j* is denoted by $p_{ij}^{k}$. *p*_*d*_ is the amount of fuel used by the car when it is fully loaded and traveling at an average speed per unit of distance. *p*_0_ is the amount of fuel used per unit of distance driven by the car when it is not carrying any weight and is moving at a regular speed. *POP*_*ij*_ is the number of people living between nodes *i* and *j*. *β*_*j*_ is the self-delivery utility index of the residential node *j*. *η*_*j*_ is the self-delivery habit bias coefficient of the household node *j*. The maximum distance *d*_max_ between each home node and the collecting center is indicated. The minimum distance *d*_min_ between each home node and the collecting center is indicated. The maximum quantity of medical waste generated by a home between its nodes is denoted by *AW*_max_. The minimum quantity of medical waste generated by a home between its nodes is denoted by *AW*_min_. The utility threshold for household nodes to deliver medical waste to collection centers on their own is represented by the symbol *ω*.

*l*_*j*_ is a 0–1 variable if node *j* delivers household medical waste to the collection center on its own, *l*_*j*_ = 1, otherwise, *l*_*j*_ = 0. $f_{j}^{k}$ denotes a 0–1 variable if the household node *j* is serviced by a collection vehicle, $f_{j}^{k}=1$, otherwise, $f_{j}^{k}=0$. $g_{ij}^{k}$ is a 0–1 variable if the collection vehicle *k* travels from node *i* to node *j*, $g_{ij}^{k}=1$, otherwise, $g_{ij}^{k}=0$. $h_{j}^{k}$ is a 0–1 variable if the collection vehicle *k* travels from the collection center to the household node *j*, $h_{j}^{k}=1$, otherwise, $h_{j}^{k}=0$.

### 3.3 Construction of vehicle velocity function

With the increase in the number of household automobiles, the frequency of road congestion increases, and even when a car accident occurs, the congestion on the road can be even worse. In the process of transporting household medical waste, there is a particular risk of contamination from medical waste leakage. Traffic congestion seriously affects the speed of household medical waste transportation, and the pollution risk of household medical waste will change with traffic congestion. To simplify the study, the road congestion coefficient *α* is introduced to react to the degree of traffic congestion, where *α*∈[0,1). *α* = 0 indicates that the traffic on the road is unimpeded. When *α* is closer to 1, it indicates that the road section is more congested. Improvements are made based on the known function for calculating the speed of vehicle travel in the case of road congestion, and the function of vehicle travel speed is obtained as shown in Eq (1).

$$v^{\prime }=(1-\alpha )\times v+(1-\frac{e^{\alpha }}{8.14})\times \alpha \times v$$ (1)

Where *v*′ denotes the collection of vehicle travel speeds in the presence of road congestion.

### 3.4 Construction of pollution risk function

Vehicles used to collect infectious medical waste usually follow a certain standard. When vehicles are used to collect infectious medical waste, they usually need to be equipped with containers made of metal or high-density plastic. These containers are usually rigid, impermeable, puncture-proof, and tamper-proof [20]. However, not all countries meet these standards due to factors such as the high cost of the container and the fact that it is not easily made. If the requirements are unmet, there is a high risk of impacting the surrounding environment while transporting contaminated medical waste in vehicles.

During the transportation of household medical waste, the risk of transportation arises mainly from the leakage of pollutants, which in turn affects the surrounding population around the transportation, and the number of pollutants inhaled by the population reflects the level of risk of contamination during the transportation process. The majority of the contaminants examined in this study are airborne. The amount of home medical waste transferred, the vehicle’s trip time, and the surrounding environmental factors all have a significant impact on how these toxins disperse. The primary analysis is on how leaking toxins move throughout the atmosphere, exposing nearby populations through inhalation or lifting. To reflect the degree of environmental hazards and the potential number of victims of the collection vehicle during transportation, this paper sets the risk of household medical waste pollution(*ERP*), which is the product of the amount of escaped pollution from household medical waste(*CON*), the number of surrounding inhabitants(*POP*), and the transportation time(*t*) during transportation, i.e.,

ERP=CON×POP×t (2)

As a result of wind speed, the rate of dissipation of escaping pollutants from household medical waste is faster than when there is no wind. The dissipation rate of pollutants increases with the increase of wind speed. Assuming that the contaminants are uniformly distributed around the collection vehicle, the amount of contamination from leaking household medical waste can be expressed by Eq (3).

$$CON_{ij}=(1-\theta_{ij})\times p_{ij}^{k}\times \gamma$$ (3)

Where *θ*_*ij*_∈[0,1] denotes the wind speed between node *i* and node *j*, and as *θ*_*ij*_ converges more to 1, then the wind speed increases. $p_{ij}^{k}$ is the amount of household medical waste loaded by the vehicle *k* as it travels between node *i* and node *j*. *γ* is the contamination rate per unit of household medical waste.

If *ξ*_*ij*_ is the population density per unit of distance in the path between node *i* and node *j*, then the total population affected at a distance *d*_*ij*_ between node *i* and node *j* is as follows:

$$POP_{ij}=\xi_{ij}\times d_{ij}$$ (4)

Substituting Eqs (3) and (4) into Eq (2), the risk of household medical waste contamination can be obtained as follows:

$$ERP=\sum_{i\in E}\sum_{j\in E}(1-\theta_{ij})\times p_{ij}^{k}\times \gamma \times \xi_{ij}\times d_{ij}$$ (5)

### 3.5 Mathematical model

In this paper, the BOMIP model is proposed for the vehicle path problem in a household medical waste recycling logistics network to determine the routes of vehicles collecting household medical waste, estimate the total cost of this logistics network, and assess the pollution impact of household medical waste. To better fit the actual transportation environment, a vehicle speed function is introduced to simulate the vehicle’s traveling speed on different road sections. The model considers two different objective functions, i.e., the total cost of the recycling logistics network and the risk of contamination. The formulas of the proposed model are given below:

(1) Total Cost of the Recycling Logistics Network

Eq (6) is the first objective function in the bi-objective mixed integer model developed in this paper, whose goal is to minimize the total cost of the network. This objective function is formulated by summing Eqs (6. a) through Eq (6. d). Eq (6. a) is the fixed cost of use incurred when a collection vehicle is dispatched.


$$Minimize\;Z_{1}=CK\times m$$ (6. a)

Eq (6. b) represents the transfer costs incurred by collection vehicles for the transfer of household medical waste.


$$+CT\times \sum_{j\in Q}AW_{j}\times (q-\sum_{j\in Q}l_{j})$$ (6. b)

Eq (6. c) is the cost of collecting the fuel consumption incurred during the operation of the vehicle.


$$+COF\times \sum_{k\in K}\sum_{i\in E}\sum_{j\in E}FC_{ij}^{k}\times d_{ij}\times g_{ij}^{k}$$ (6. c)

Eq (6. d) is the incentive received by the household node for taking household medical waste to the collection center on its own.


$$+PR\times \sum_{j\in Q}AW_{j}\times l_{j}$$ (6. d)

$FC_{ij}^{k}$ in Eq (6. c) is the amount of fuel consumed per unit of distance traveled by collection vehicle *k* from node *i* to node *j*, calculated as shown in Eq (7).


$$FC_{ij}^{k}=(|\frac{v-v_{ij}^{'}}{v}|+1)(\frac{p_{d}-p_{0}}{D}p_{ij}^{k}+p_{0})$$ (7)

(2) Pollution Risk

The second objective function of the model is represented by Eq (8), which aims to minimize the impact of pollution risks on the surrounding population.


$$Minimize\;Z_{2}=ERP$$ (8)

The model is subject to:

$$\sum_{i\in E}g_{ij}^{k}=\sum_{l\in E}g_{jl}^{k}\;\forall k\in U,\forall j\in E,i\neq j,j\neq l$$ (9)

Constraint (9) is a constraint on the continuity of the vehicle path.


$$\sum_{k\in U}f_{j}^{k}=1\;\forall j\in M$$ (10)

Constraint (10) implies that a household node that recycles via a collection vehicle is served by one and only one collection vehicle.


$$\sum_{j\in M}g_{ij}^{k}=f_{i}^{k}\;\forall i\in M,\forall k\in U$$ (11)

$$\sum_{i\in M}g_{ij}^{k}=f_{j}^{k}\;\forall j\in M,\forall k\in U$$ (12)

Constraints (11) and (12) ensure that household nodes for recycling via collection vehicles can only exist on one transportation route.


$$\sum_{j\in M}\sum_{k\in U}f_{j}^{k}=q-\sum_{i\in Q}l_{i}$$ (13)

Constraint (13) ensures that household nodes that are recycled through collection vehicles are served by collection vehicles.


$$\sum_{i\in M}g_{ih}^{k}-\sum_{j\in M}g_{hj}^{k}=0\;\forall h\in M,\forall k\in U$$ (14)

Constraint (14) ensures that only one home node or collection center is connected to a home node both before and after it in the travel path formed by the collection vehicle.


$$\sum_{j\in M}h_{j}^{k}\leq u\;\forall k\in U$$ (15)

Constraint (15) guarantees that the number of collection vehicles dispatched from the collection center does not exceed the total number of collection vehicles.


$$\sum_{j\in M}\sum_{k\in U}g_{ij}^{k}\geq 1\;\forall i\in P$$ (16)

Constraint (16) ensures that at least one collection vehicle departs from the collection center.


$$\sum_{i\in P}\sum_{j\in M}g_{ij}^{k}\leq 1\;\forall k\in U$$ (17)

Constraint (17) indicates that each collection vehicle departs from the collection center at most once.


$$\sum_{j\in M}AW_{j}\times f_{j}^{k}\leq D\;\forall k\in U$$ (18)

Constraint (18) ensures that the amount of household medical waste loaded on the collection vehicle does not exceed the maximum load capacity of the vehicle.


$$\sum_{j\in M}g_{ij}^{k}=\sum_{j\in M}g_{ji}^{k}\;\forall i\in P,\forall k\in U$$ (19)

Constraint (19) ensures that the collection vehicle departs from the collection center and returns to the collection center upon completion of the collection task.


$$v_{ij}^{'}=(1-\alpha_{ij})\times v+(1-\frac{e^{\alpha_{ij}}}{8.14})\times \alpha_{ij}\times v$$ (20)

Constraint (20) calculates the speed of travel between two nodes of the collected vehicles in case of road congestion.


$$t_{ij}=\frac{d_{ij}}{v_{ij}^{'}}$$ (21)

Constraint (21) calculates the time consumed to collect vehicles traveling between two nodes.


$$\sum_{i\in E}\sum_{j\in E}d_{ij}\times g_{ij}^{k}\leq L\;\forall k\in U$$ (22)

Constraint (22) indicates that the total distance traveled by the collection vehicle does not exceed the maximum range of the vehicle.


$$\beta_{j}=\eta_{j}\times \frac{d_{\max}-d_{ij}}{d_{\max}-d_{\min}}+(1-\eta_{j})\times \frac{AW_{j}-AW_{\min}}{AW_{\max}-AW_{\min}}$$ (23)

Constraint (23) calculates the self-delivery utility metric of household nodes to quantify the willingness of household nodes to self-deliver medical waste to collection centers.


$$l_{j}=\{\begin{array}{l} 1\;\beta_{j}\geq \omega \\ 0\;\beta_{j}<\omega \end{array}$$ (24)

Constraint (24) determines whether the household node sends the household medical waste to the collection center by itself.

## 4. Solution approach

The vehicle path problem is known to have the NP-hard property alone, which means that finding an optimal solution to the large-scale problem is complex [66]. In addition, the two objective functions of the proposed model in this study contradict each other, so it is reasonable to use a MO-meta-heuristic algorithm to solve the problem. Therefore, in this paper, we develop an adaptive hybrid artificial fish swarming algorithm with non-dominated ordering, which differs from the classical artificial fish swarming algorithm in several aspects.

### 4.1 Representation scheme for solutions

The choice of solution representation scheme is one of the important factors that affect the complexity and required computational time of an intelligent optimization algorithm. Using a proper solution representation scheme can guide the algorithm to find more likely candidate solutions. We used the widely used natural number-coding method for artificial fish. If the number of generating nodes is *L* and the number of transit vehicles is *K*, the coding length at this point is *L*×*K*, and the coding is done by randomizing all the integers in [1,*L*×*K*]. Then the form of the code can be expressed as [*X*_1_,*X*_2_,*X*_3_,⋯,*X*_*L*×*K*_], where *X*_*j*_ represents the number whose position index is *j* in the code. If there are four generating nodes and two transshipment vehicles, the code for one possible artificial fish is [3,5,7,8,1,2,6,4].

The decoding process converts the representation of the solution into a meaningful solution and obtains the objective function value. By decoding the artificial fish, it is possible to determine which generating node should be provided with collection service by which transit vehicle and it is possible to derive the order in which each transit vehicle provides collection service to the generating node. It is known that the representation of the artificial fish can be obtained by encoding the natural numbers [*X*_1_,*X*_2_,*X*_3_,⋯,*X*_*L*×*K*_]. The generating node *i* corresponding to the position located at the position *j* in the encoding and the collection vehicle *m* providing collection service to this node can be calculated by using Eqs (25) and (26).

$$i=S_{j}-\lfloor \frac{S_{j}-1}{L}\rfloor \times L$$ (25)

$$m=\lfloor \frac{S_{j}-1}{L}\rfloor +1$$ (26)

Where ⌊⌋ is denoted as rounding down. At this point, the generating node and the transshipment vehicle corresponding to each position in the artificial fish can be obtained. The specific flow of decoding is shown in S1 Fig., which yields which transshipment vehicle should perform the collection service for each generating node and the collection order of each vehicle for the generating node.

### 4.2 Artificial fish swarming algorithm

The Artificial Fish Swarming Algorithm (AFSA) proposed by Li et al. is a swarm intelligence optimization algorithm that simulates the behaviors of fish, such as foraging, aggregation, and tail chasing [67]. In AFSA, fish often gather in the vicinity of waters that contain large amounts of nutrients. The artificial fish swarming algorithm simulates the behaviors of foraging, tail-chasing, and swarming based on the fish’s ability to gravitate toward food and avoid dangers to achieve the goal of optimization. Each artificial fish performs swarming behavior as per (29) and tail-chasing behavior as per (30), depending on its current environment. If no improvement is obtained, the foraging behavior is performed according to Eq (31). If there is still no improvement, random behavior is performed according to Eq (32).

$$X_{j}=X_{i}+visual\times Rand()$$ (27)

$$X_{c}=\frac{\sum_{j=1}^{n_{f}}X_{j}}{n_{f}}$$ (28)

$$X_{next}=X_{i}+\frac{X_{c}-X_{i}}{\left\|X_{c}-X_{i}\right\|}\times step\times Rand()$$ (29)

$$X_{next}=X_{i}+\frac{X_{\max}-X_{i}}{\left\|X_{\max}-X_{i}\right\|}\times step\times Rand()$$ (30)

$$X_{next}=X_{i}+\frac{X_{j}-X_{i}}{\left\|X_{j}-X_{i}\right\|}\times step\times Rand()$$ (31)

$$X_{next}=X_{i}+step\times Rand()$$ (32)

Wherein Eq (27) indicates that the positional alternation is performed, i.e., the artificial fish *X*_*j*_ within the current field of view is randomly selected, and Eq (28) calculates the center position of all the artificial fish within the current perceptual range. Where *X*_*i*_ denotes the current position of the artificial fish *i*, *X*_*j*_ denotes the position of the artificial fish *j* within the field of view, *Rand*() is the random number of (0,1) within the interval, *n*_*f*_ denotes the number of all artificial fish within the field of view of the artificial fish *j*, and *X*_*next*_ denotes the new better position of the artificial fish achieved after performing positional updating.

The artificial fish swarm algorithm has the advantages of robustness, parallelism, global search ability, and low requirements on the initial value and the nature of the objective function. However, the algorithm also has shortcomings such as poor optimization accuracy, easy fall into local extremes, and slow convergence speed.

### 4.3 Adaptive Hybrid Artificial Fish Swarming method non-dominated sorting

This section describes the modifications to the artificial fish swarming algorithm in this study. An Adaptive Hybrid Artificial Fish Swarming Algorithm with Non-Dominated Sorting (AH-NSAFSA) is proposed to address the shortcomings of the artificial fish swarming algorithm and the optimization search for bi-objectives.

(1) Adaptive Improvement

One of the modifications occurred in the expression of the field of view, step size, and crowding factor of the artificial fish. In the Artificial Fish Swarming Algorithm, the field of view, step size, and crowding factor are all essential parameters that can have a direct impact on the performance of the algorithm. Therefore, a decay function *ρ*, which varies with the number of iterations of the algorithm as expressed in Eq (33), is introduced into the expression of the parameters so that the field of view, step length, and crowding factor can be changed adaptively with the operation of the algorithm, as follows:

$$\rho =\sqrt[{d_{\max}-d+1}]{\exp(-\frac{d^{4}}{d_{\max}^{3}})}$$ (33)

$$step=\lfloor \rho \times step_{0}+(1-\rho )\times step_{\min}\rfloor$$ (34)

$$visual=\lfloor \rho \times visual_{0}+(1-\rho )\times visual_{\min}\rfloor$$ (35)

$$\delta =\lfloor \rho \times \delta_{0}\rfloor$$ (36)

Where *d*_max_ and *d* denote the maximum number of iterations of the algorithm and the number of generations the algorithm is currently in, respectively. *step*_0_ and *step*_min_ denote the initial step size and the minimum step size, respectively. *visual*_0_ and *visual*_min_ refer to the initial field of view as well as the minimum field of view. *δ*_0_, on the other hand, denotes the initial congestion factor.

(2) Non-dominated Sorting Improvement

Deb et al. proposed the technique of non-dominated sorting to find the non-dominated solution for each iteration [68]. In the technique of non-dominated sorting, each artificial fish is compared with the other individuals in the population, which in turn yields the Pareto frontier of non-dominated sorting. A MO-optimization problem is a set of maximization or minimization objectives defined in the solution space. For the proposed model with two minimization objectives, a solution *y* is said to be dominated by another solution *x* if, for all objectives, we have at least one objective satisfied *OFV*_*obj*_(*y*)<*OFV*_*obj*_(*x*). This means that the solution *x* dominates the solution *y* if the value of the objective function of the solution *y* is less than or equal to the value of the objective function of the solution *x*, and at least one of the values of the objective function of the solution *y* is less than the value of the objective function of the solution *x*. Domination ordering divides the population into different levels, and the same comparison is required between the dominating solutions. For this purpose, the crowding distance, i.e., the density of individuals around a given point in the population, is used, which indicates the smallest rectangle around a given individual that contains the individual itself but no other individuals. The population is subjected to the calculation of non-dominated ordering and crowding distance, and each individual in the population receives the attributes of non-dominated ordering and crowding distance. If two individuals have different non-dominated orderings, the individual with the smaller ordinal number is taken. If two individuals are at the same level, the less crowded individual is taken.

(3) Hybrid Improvement

In addition to the above modifications, to ensure the overall quality of the population, this paper draws on the crossover operation in the genetic algorithm to design an elimination behavior. That is, the worst two artificial fish are eliminated, and the fitness values of the optimal two artificial fish are used to generate two new artificial fish through the worst operation of the genetic algorithm. When the algorithm proceeds to the middle and late stages, after every certain number of iterations, all the artificial fish are sorted according to their nondominant ordering and crowding distance, and the worst two artificial fish are eliminated from the process. The two optimal artificial fish are used to generate two new artificial fish to replace the eliminated ones through the OX crossover operation. The OX crossover is performed as follows:

**Step 1**: The starting and ending positions of the two artificial fish codes were randomly selected, and the selected positions were the same for both artificial fish.

If two artificial fish individuals are:

FISH112345678

FISH225476813

At this point, the randomly selected starting and ending positions are *a* = 3 and *b* = 6, and the segments of the crossover are:

FISH112|345|678

FISH225|476|913

**Step 2**: Mutual copying of cross-segments. That is, the cross-segment of Artificial *FISH*1 is copied to the front of Artificial *FISH*2, and the cross-segment of Artificial *FISH*1 is copied to the front of Artificial *FISH*2. At this point, the Artificial Fish becomes:

FISH147612345678

FISH234525476813

**Step 3**: Duplicate-coded spots were marked from front to back, and two new artificial fish could be obtained by removing the second duplicate spot from the process.


New−FISH147612358

New−FISH234527681

S2 Fig. shows the flowchart of the proposed algorithm.

## 5. Computational experiment

This section provides numerical experiments to evaluate the performance of the algorithm. The vehicle path problem is an NP-hard problem and is very complex to solve. Its complexity increases when the number of generating nodes, collection centers, and transit vehicles is significant. Therefore, using the swarm intelligent optimization algorithm to solve the model proposed in this paper is reasonable. We coded the algorithms using MATLAB software (R2023b) and ran the methods on an Intel Core i9 personal computer (2.20 GHz CPU) with 16 GB of memory (RAM).

To evaluate the performance of AH-NSAFSA and compare it with the Artificial Fish Swarming Algorithm with Non-Dominated Sorting (NSAFSA), a test problem with 31 nodes was randomly selected from RC101 of the Solomon dataset. This includes one collection center and 30 generator nodes. The program automatically generates the road congestion coefficient, wind speed, population density, and self-feeding habit bias coefficients of each node in the model. Other relevant parameter settings are shown in Table 1.

**Table 1:** Time-sharing tariff.

Parameter	Parameter values	Parameter	Parameter values
*u*	8 cars	*COF*	¥0.12/L
*D*	100kg	*PR*	¥2/kg
*L*	700km	*p*_0_	10L/km
*v*	20km/h	*p*_*d*_	20L/km
*CK*	¥50/car	*γ*	0.3
*CT*	¥1/kg	*ω*	0.7

### 5.1 Parametric calibration

The values of the parameters controlled by the metaheuristic algorithm can affect its optimization search performance [68]. Therefore, in this study, the control parameters of AH-NSAFSA are calibrated by the Taguchi Method (*TM*), which in turn improves the algorithm’s optimization search efficiency. By using the Taguchi method, a large amount of information can be obtained by conducting a minimum number of experiments [69]. In this study, a five-level Taguchi design is considered for the parameters of AH-NSAFSA. To avoid errors caused by different measures, the objective function values are normalized. Then, the normalized use of the objective for the solution *j* ($NOFV_{j}^{1}$ and $NOFV_{j}^{2}$) is obtained through Eqs (37) and (38).


$$NOFV_{j}^{1}=\frac{OFV_{1}^{*}}{OFV_{j}^{1}}$$ (37)

$$NOFV_{j}^{2}=\frac{OFV_{2}^{*}}{OFV_{j}^{2}}$$ (38)

Where $OFV_{j}^{1}$ and $OFV_{j}^{2}$ denote the first objective function value and the second objective function value in the solution *j*, respectively. The optimal function values for the first and second objectives are denoted by $OFV_{1}^{*}$ and $OFV_{2}^{*}$, respectively. For the solution *j*, the total objective function value (*TOFV*_*j*_) is calculated from Eq (39).

$$TOFV_{j}=\frac{1}{\theta \times NOFV_{j}^{1}+(1-\theta )\times NOFV_{j}^{2}}$$ (39)

Where *θ*(0≤*θ*≤1) is denoted as the relative importance of the two objective functions, this study is based on the idea of human-centeredness, so *θ* takes the value of 0.4. Next, the total objective function value is converted into a relative percentage deviation (*RPD*) by using Eq (40) [70].

$$RPD=\frac{\left|TOFV_{j}-BOFV\right|}{BOFV}\times 100$$ (40)

$$S/N=-10\times \log(\sum \left(RPD^{2}\right)/n)$$ (41)

**Fig 2:**
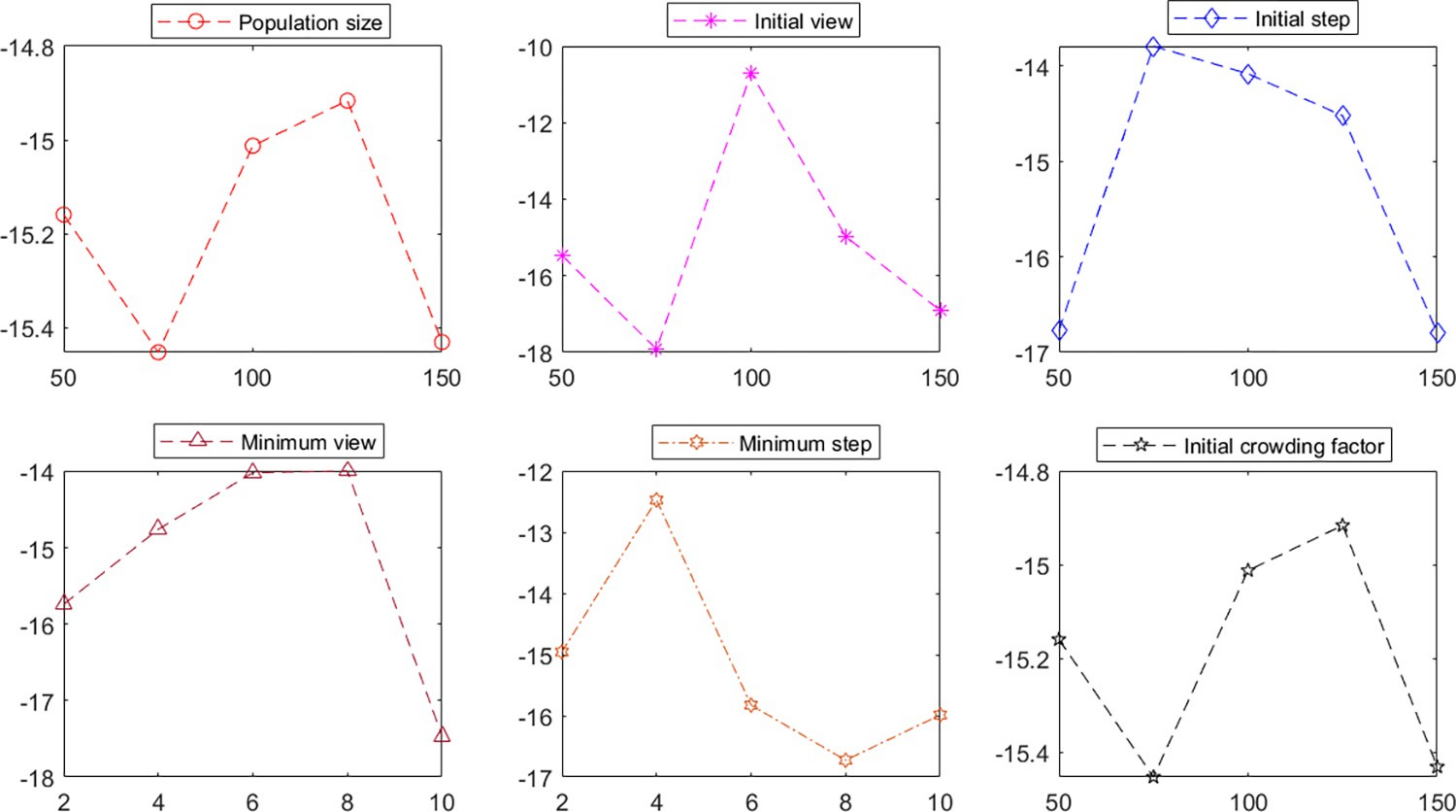
The S/N ratio plot of the AH-NSAFSA in the Taguchi methodology.

Where *BOFV* is the best of the total objective function values, the parameter levels that have been considered are shown in Table 2. The signal-to-noise ratio (*S*/*N*) is calculated through Eq (41). A more significant value of the signal-to-noise ratio represents a better quality of the control parameter. Based on the maximum signal-to-noise ratio of each control parameter, the optimum level of each control parameter is selected. Then, the selected parameter levels are shown in Table 3. In addition, Fig 2 depicts the average *S*/*N* plot for the test problem.

**Table 2:** Levels of input parameters.

Parameter	Level 1	Level 2	Level 3	Level 4	Level 5
Population size	50	75	100	125	150
Initial view	75	70	65	60	55
Minimum view	10	8	6	4	2
Initial step	75	70	65	60	55
Minimum step	10	8	6	4	2
Initial crowding factor	1	0.8	0.6	0.4	0.2

**Table 3:** Tuned values of parameters.

Parameter	Values
Population size	125
Initial field of view	65
Minimum field of view	8
Initial step	60
Minimum step	4
Initial crowding factor	1

### 5.2 Performance evaluation metrics

The literature related to MO-optimization problems provides numerous metrics to evaluate the performance of MO-optimization algorithms. Five of these metrics are used in this study to evaluate the performance of AH-NSAFSA and NSAFSA in solving the test problems. A description of the metrics is provided inS1 File.

### 5.3 Analysis of results

In this section, to validate the ability of AH-NSAFSA to solve the proposed model, we use two algorithms, the Non-dominated Sorted Artificial Fish Swarm Algorithm (NSAFSA) and the Non-dominated Sorted Genetic Algorithm II (NSGA-II), for comparison. Each algorithm was run 10 times on the test algorithm, and Table 4 lists the average values of CT, MID, SM, DM, NOS, ATA, and ATO for each of the 10 runs. An analysis of the data results leads to the following conclusions:(1) In terms of the number of non-dominated options. AH-NSAFSA produces a higher number of non-dominated options, which can provide a wider range of alternatives to the decision-maker. This is evidenced by the higher NOS values, which are 4% higher for AH-NSAFSA than for NSAFSA and 73.3% higher than for NSGA-II. (2) Metrics such as MID, SM, and DM are not applicable here due to the limited number of non-dominated scenarios obtained by NSGA-II. (3) The SM value reflects the consistency of the algorithm’s non-dominated solutions, and a smaller value indicates a more consistent solution. The SM value of AH-NSAFSA is 19.6% less than the SM value of NSAFSA. It indicates that AH-NSAFSA outperforms NSAFSA in this dimension. (4) The search efficiency of the algorithm is expressed by the MID value, and the smaller value of MID indicates that the algorithm is more efficient in searching. The value of AH-NSAFSA is 46.9% lower than NSAFSA in terms of MID. Therefore, the search efficiency of AH-NSAFSA is better than NSAFSA. (5) When considering the generation of more diversified solution sets, as reflected by the DM value, a higher DM value indicates a more diversified set of solutions generated. The DM value of AH-NSAFSA is 14.7% lower than the DM value of NSAFSA. Therefore, AH-NSAFSA does not perform as well as NSAFSA in terms of solution diversity. (6) The CPU occupancy time of NSGA-II in identifying individual solutions (ATO metrics) and the entire set of solutions (ATA metrics) is much higher than that of AH-NSAFSA and NSAFSA. (7) In terms of metrics ATO, the CPU occupancy time of AH-NSAFSA is 13.4% less than that of NSAFSA by 13.4%. On the metric ATA AH-NSAFSA takes 9.9% less CPU time than NSAFSA. Therefore, whether searching for a single solution or the whole solution set, AH-NSAFSA has a better search speed than NSAFSA.

**Table 4:** Comparison of algorithms across metrics in test problems.

Algorithm	CT	MID	SM	DM	NOS	ATA	ATO
AH-NSAFSA	1507.0750	94722.9618	108980.0323	173578.6395	2.6	1507.0750	579.6442
NSAFSA	1672.9965	178281.942	135625.9435	203410.1441	2.5	1672.9965	669.1986
NSGA-Ⅱ	52.5689	/	/	/	1.5	52.5689	35.046

A statistical estimation of the performance of the algorithms was done to better compare the performance of the algorithms. The 2-sample t-test and the following hypotheses were used for statistical comparisons. These comparisons were made based on each performance metric at a confidence level of 95%. The original hypothesis (*H*_0_) assumes that there is no significant difference in the values of the performance evaluation metrics for each algorithm. The alternative hypothesis (*H*_1_) assumes the opposite. Therefore, the original hypothesis (*H*_0_) is rejected if the p-value is less than 5% [71].

*H*_0_: There is no significant difference in the evaluation metrics of the algorithms.

*H*_1_: There is a significant difference in the evaluation metrics of the algorithms.

The two algorithms have solved ten test problems. The comparison between the algorithms in terms of the performance metrics MID, SM, DM, CT, and NOS is discussed below.

Table 5 delineates a comparative evaluation of the performance indices of the two algorithms in question: the AH-NSAFSA and the NSAFSA. The statistical findings presented in Table 5 demonstrate that the p-values for the MID and CT metrics are below the 5% significance threshold. This observation leads to the rejection of the null hypothesis (*H*_0_) for these metrics, signifying a statistically significant difference between the algorithms. Specifically, it underscores that AH-NSAFSA outperforms NSAFSA in terms of optimization efficacy and computational expedience. Conversely, the p-values for the SM, DM, and NOS do not surpass the 5% threshold, indicating that the null hypothesis remains tenable for these metrics. This suggests that there is no statistically discernible disparity in performance between AH-NSAFSA and NSAFSA concerning the aspects above. The implication is that AH-NSAFSA does not exhibit a significant shortfall in comparison to NSAFSA concerning the provision of a diverse and uniformly distributed set of non-dominated solutions. In light of these findings, it is concluded that the proposed AH-NSAFSA algorithm is efficacious in addressing the problem at hand, offering a robust optimization framework that is particularly distinguished by its superior convergence speed and overall computational performance.

**Table 5:** ANOVA was performed to test the differences between the algorithms for each indicator.

Performance measure	Combined variance	t State	p-Value	Result
MID	6.15E+09	-2.3817	0.02847	*H*_0_ is rejected
SM	1.69E+10	-0.4585	0.65208	*H*_0_ is accepted
DM	3.22E+10	-0.3716	0.71456	*H*_0_ is accepted
CT	7826.8854	-4.1937	0.000546	*H*_0_ is rejected
NOS	0.366667	0.7385	0.46970	*H*_0_ is accepted

## 6. Managerial insights

This study provides innovative bi-objective modeling on an academic and practical level, providing valuable insights and implications for managers when dealing with the household medical waste recycling pathway problem. Below are some key points outlining the potential implications of this study for managers:

1. A balanced decision-making framework: The study suggests that a decision-making framework considering both operating costs and pollution risks is more comprehensive and practical than one seeking to minimize costs. This balanced approach helps companies balance financial efficiency and environmental responsibility.
2. Green Lifestyle Advocacy: Against society’s increasing emphasis on green living and environmental awareness, pollution risk management in the logistics network is crucial to a company’s public image. A company’s environmental performance directly affects its perception of its customers, which in turn affects its reputation, customer loyalty, and market penetration.
3. Customized collection options: Companies should choose the most appropriate waste collection option based on their operational experience, regional demographics, environmental conditions, and local government policy requirements. This customized approach ensures that the business meets regulatory requirements while responding effectively to the challenges of the particular environment.
4. Risk assessment and management: The study highlights the importance of assessing and minimizing the risk of contamination when planning and implementing waste recycling routes. Companies need to develop and implement effective risk management strategies to minimize waste transport’s potential environmental and community impacts.
5. Application of technology and innovation: The AH-NSAFSA algorithm proposed in this study demonstrates how advanced optimization techniques can solve complex waste recycling routing problems. Managers can learn from this and explore how similar techniques can be applied to other operational challenges.
6. Performance evaluation and continuous improvement: Through a rigorous evaluation of the algorithm’s performance, elucidated a framework for the empirical validation of solution efficacy. This systematic methodology is a paradigm for managers to emulate in their operational practices. By instituting a regimen of periodic performance assessments and subsequent optimization of waste management protocols, they can ensure a trajectory of perpetual enhancement. Such a dynamic approach facilitates adapting to evolving conditions and refining strategies in response to new challenges and opportunities within the domain of waste management.
7. Stakeholder communication: Companies should communicate effectively with customers, community members, government agencies, and other stakeholders to convey their efforts and achievements in reducing the risk of contamination in the waste recycling process. This transparency helps build trust and support.
8. Community participation and education: Increasing public awareness and participation in properly disposing of household medical waste can reduce improper disposal and environmental pollution.

## 7. Conclusions

### 7.1 Findings

In this paper, a multi-objective household medical waste recycling vehicle routing problem is investigated and various display factors are considered, such as the pollution impact on the environment during the transfer of medical waste, road congestion, and self-delivery of household medical waste to the recycling center. In addition to economic considerations, environmental pollution risk is also considered as an optimization objective of the model. In this paper, the environmental pollution risk was assessed through the household medical waste pollution risk function, and the effects of the amount of leaked pollution from household medical waste, the number of residents in the surrounding area, the transport time, and the wind speed on the environmental pollution risk were taken into account. Different vehicle speeds not only affect the fuel consumption of the vehicle but also influence the pollution risk of household medical waste. To better simulate the different speeds of vehicles on different roads due to congestion on urban roads, this paper introduces the road congestion coefficient to reflect the degree of congestion on urban roads. In addition, to solve the multi-objective household medical waste recycling vehicle path problem, an adaptive hybrid artificial fish swarm algorithm with non-dominated sorting (AH-NSAFSA) is proposed. The adaptive iteration mechanism, by introducing a decay function, enables the algorithm to dynamically adjust the search strategy during the iteration process, thus improving the adaptability and search efficiency of the algorithm. The elimination behavior, which can better enable the population to maintain a high-quality level during the search process, guarantees the quality of the algorithm solution. The effectiveness of AH-NSAFSA is verified through numerical experiments and compared with two existing state-of-the-art algorithms: the Non-dominance Sorted Artificial Fish Swarm Algorithm (NSAFSA) and the Non-dominance Sorted Genetic Algorithm II (NSGA-II) in terms of a variety of performance evaluation indexes. The experimental results are summarized as follows.

1. AH-NSAFSA can better solve the multi-objective household medical waste recycling pathway problem considering pollution risk and can provide multiple non-dominated solutions to the decision maker for selection.
2. AH-NSAFSA is compared with NSAFSA and NSGA-II and judged by the evaluation metrics of multiple multi-objective optimization algorithms of CT, MID, SM, DM, NOS, ATA, and ATO. The results show that AH-NSAFSA has better search efficiency and consistency of non-dominated solutions, and produces a larger number of non-dominated solutions. Due to the introduction of an adaptive iteration mechanism and elimination behavior, AH-NSAFSA has better speed than NSAFSA in searching for a single solution and the whole solution set.
3. The model and algorithm proposed in this paper can effectively plan vehicle paths for household medical waste recycling.

### 7.2 Limitations and recommendations for future research

In this study, a two-objective mixed-integer planning model was developed by combining both total cost and pollution risk as objectives and considering road congestion and self-delivery of household medical waste to recycling centers. The Pareto optimal solution obtained through AH-NSAFSA helps to provide data support for the decision maker’s choice and is more conducive to the sustainable forward development of the household medical waste recycling industry. The model developed in this paper is deterministic, however, there are many uncertainties in household medical waste management services, such as fluctuations in waste volume, changes in traffic conditions, unpredictability of weather conditions, etc. In the next study, incorporating uncertainty into the model parameters may improve the adaptability and robustness of the model. This may involve probabilistic modeling, fuzzy logic, or scenario analysis to better capture and reflect these uncertainties. Secondly, the model presented in this paper is static. Whereas the amount of household medical waste generated and the collection demand may change during the household medical waste recycling process, these real-time changes can be accommodated by dynamic modeling in the future to further improve the efficiency and effectiveness of waste management. In addition, more in-depth research can be conducted on the solution effect and solution efficiency of the optimization solution algorithm, and more optimization algorithms or the combination of machine learning and optimization algorithms can be used to solve the problem in the future. Finally, accidents such as traffic accidents may be encountered during the recycling and transport of household medical waste, and these accidents may lead to the leakage of waste, which in turn increases the risk of environmental pollution and public health. In case of accidents, how to re-plan the path of recycling vehicles to reduce the risk of pollution. And these accidents can be considered in future research.

## Supporting information

S1 Fig Decoding process. (TIF)

S2 Fig Flowchart of the AH-NSAFSA. (TIF)

S1 Table Summary of the most related studies. (DOCX)

S1 File Description of performance assessment indicators. (DOCX)
