# Supplementary figures and images for: Optimization of household medical waste recycling logistics routes: Considering contamination risks
Source: PLoS One. 2024 Oct 7;19(10):e0311582. doi: 10.1371/journal.pone.0311582 (PMC11458020; doi:10.1371/journal.pone.0311582)

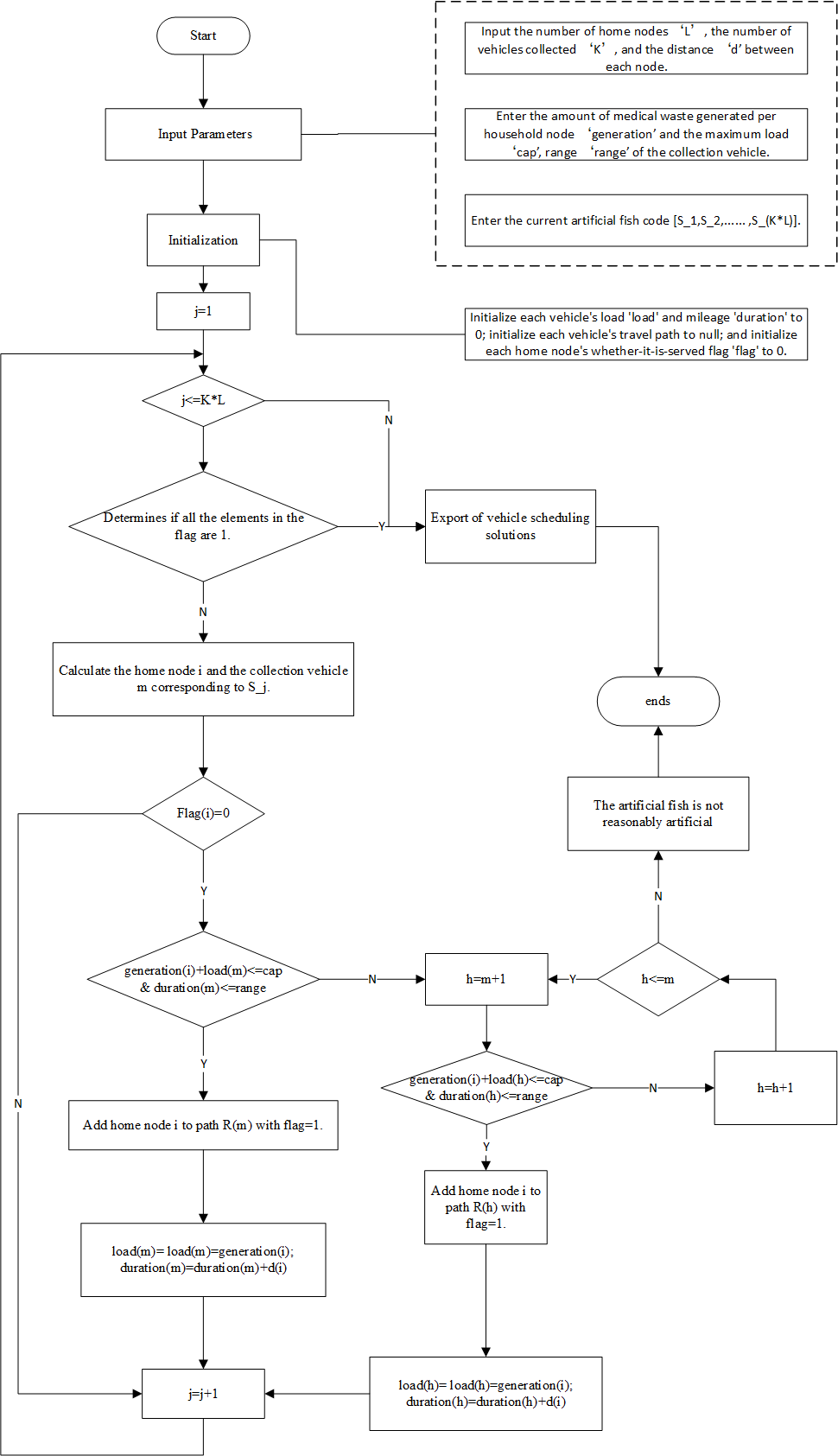

Supplement: S1 Fig — (TIF) [file pone.0311582.s001.tif]

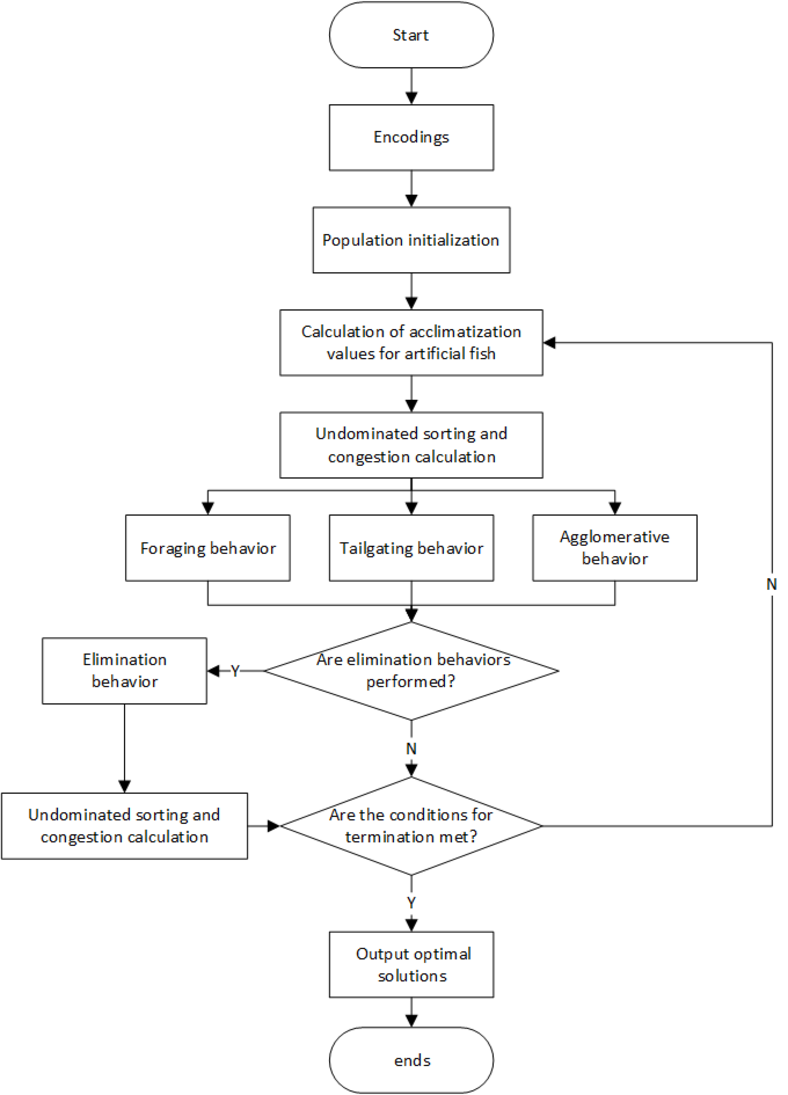

Supplement: S2 Fig — (TIF) [file pone.0311582.s002.tif]
