# Supplementary material for: Optimization of household medical waste recycling logistics routes: Considering contamination risks
Source: PLoS One. 2024 Oct 7;19(10):e0311582. doi: 10.1371/journal.pone.0311582 (PMC11458020; doi:10.1371/journal.pone.0311582)
Supplement: S1 Table — (DOCX) [file pone.0311582.s003.docx]

**S1 Table. Summary of the most related studies.**

| Research | Problem | | | | | | | | Algorithm |
| --- | --- | --- | --- | --- | --- | --- | --- | --- | --- |
|  | Multi-objective | | Road congestion | | Pollution risk | | Household medical waste | |  |
|  | Yes | No | Yes | No | Yes | No | Yes | No |  |
| SHI Li-hong (2009) |  | * |  | * |  | * |  | * | Lingo |
| Wang et al. (2019) | * |  |  | * | * |  |  | * | Grey GM (1,1) + Lingo |
| Nikzamir et al. (2020) | * |  |  | * | * |  |  | * | MOWFA + MOCIA + MOSA |
| Govindan et al. (2022) | * |  |  | * | * |  |  | * | AUGMECON2 |
| Singh et al. (2023) | * |  |  | * |  | * |  | * | MADM + GBWM + TOPSIS + NSGA II + NSGA III + $\varepsilon$-constraint |
| Wang et al. (2023) | * |  |  | * | * |  |  | * | $\varepsilon$-constraint method |
| Nasreddine et al. (2023) |  | * |  | * |  | * |  | * | AGATE + CPLEX |
| Lin et al. (2023) | * |  |  | * | * |  |  | * | NSGA + NSGA II + MOEA/D + MOEA/D-LNS |
| Hajer et al. (2023) |  | * |  | * |  | * |  | * | EASTEP + CPLEX |
| Pereira et al. (2017) |  | * |  | * |  | * | * |  | systematic review |
| Zhang et al. (2024) | * |  |  | * | * |  |  | * | ε-constraint method + NSGA II + Compare-choose-move Algorithm |
| Cao et al. (2023) |  | * |  | * | * |  |  | * | KKT + branch-and-bound + CPLEX |
| Karimi et al. (2024) | * |  |  | * | * |  |  | * | Goal programming + TH approach + Lp-metric + GA |
| Keyvan et al. (2024) |  | * | * |  |  | * |  | * | K-means + MWPC + TSP + GIS |
| this paper | * |  | * |  | * |  | * |  | AH-NSAFSA + NSAFSA + NSGA-II |
