# Supplementary material for: Optimization of household medical waste recycling logistics routes: Considering contamination risks
Source: PLoS One. 2024 Oct 7;19(10):e0311582. doi: 10.1371/journal.pone.0311582 (PMC11458020; doi:10.1371/journal.pone.0311582)
Supplement: S1 File — (DOCX) [file pone.0311582.s004.docx]

**Description of Performance Assessment Indicators**

(1) Number of Non-dominated Solutions (NOS)

An important metric for evaluating MO-optimization algorithms is the number of non-dominated solutions on the Pareto front in the obtained results. When the number of non-dominated solutions in the obtained result is higher, it means that there are more options available to the decision-maker in the problem ^[1]^.

(2) Mean Ideal Distance (MID)

The Mean Ideal Distance (MID) measures the distance between the non-dominated solution of the Pareto front of the algorithm and the ideal point. The smaller the value of MID, the better the algorithm's performance. This metric is calculated by using equation (C. 1) ^[2]^.

$MID=\frac{\sum_{i=1}^{NOS} \sqrt{(OF_{j}^{1}-OF_{1}^{*})^{2}+(OF_{j}^{2}-OF_{2}^{*})^{2}}}{NOS}$ (C. 1)

Where$NOS$ is the number of non-dominated solutions. $OF_{j}^{1}$ and$OF_{j}^{2}$ denote the values of the first objective function and the second objective function for the solution$j$, respectively. $OF_{1}^{*}$ and$OF_{2}^{*}$ denote the values of the first and second objective functions for the ideal point, respectively.

(3) Spacing Metric (SM)

This metric is used to characterize the distribution of the non-dominated solutions of the resulting Pareto front. When the value of SM is lower, it means that the solutions obtained by the algorithm are more evenly distributed. SM can be obtained by using equation (C. 2) ^[3]^.

$SM=\sqrt{\frac{1}{NOS-1}\sum_{i=1}^{NOS} (D_{i}-\bar{D})^{2}}$ (C. 2)

$D_{i}=\min_{i'} \{\left|OF_{i}^{1}-OF_{i'}^{1}\right|+\left|OF_{i}^{2}-OF_{i'}^{2}\right|\}\begin{matrix} & i,i'=1,2,\ldots,NOS \end{matrix}$ (C. 3)

$\bar{D}=\sum_{i=1}^{NOS} \frac{D_{i}}{NOS}$ (C. 4)

Where$D_{i}$ denotes the Euclidean distance between the dominated solution$i$ and the other solutions in the Pareto front. $D_{i}$ is obtained by the calculation of Eq. (C. 3). $\bar{D}$ is the average of all$D_{i}$ and can be obtained by Eq. (C. 4).

(4) Diversification Metric (DM)

The diversification metric is meant to characterize the diversity of solutions. When the value of DM is higher, it means that the obtained solution is more diverse. The diversity indicator can be obtained using equation (C. 5) ^[4]^.

$DM=\sqrt{(\max_{i=1:NOS} OF_{i}^{1}-\min_{i=1:NOS} OF_{i}^{1})^{2}+(\max_{i=1:NOS} OF_{i}^{1}-\min_{i=1:NOS} OF_{i}^{1})^{2}}$ (C. 5)

(5) Computing Time (CPU TIME)

The computational time metrics, in addition to assessing the efficiency of the algorithm's speed using the time used by the algorithm to find the solution (CT), were used to evaluate the merits of the algorithm's computational time using the calculation proposed by Li et al ^[5]^. The average computational time to find one solution and the average computational time to find all solutions are denoted by$ATO$ and$ATA$, respectively, and are obtained by using Eqs. (C. 6) and (C. 7).

$ATO=\frac{ATA}{\sum_{rn=1}^{RN} \frac{NOS_{rn}}{RN}}$ (C. 6)

$ATA=\frac{\sum_{rn=1}^{RN} Cotime_{rn}}{RN}$ (C. 7)

Where$RN$ is the number of runs of the algorithm. $Cotime_{rn}$ denotes the computation time of the algorithm at the $rn$th run. and$NOS_{rn}$ denotes the number of non-dominated solutions on the Pareto front obtained by the algorithm at the $rn$th run.

# References

1. Pargar F, Zandieh M, Kauppila O, Kujala J. The Effect of Worker Learning on Scheduling Jobs in a Hybrid Flow Shop: A Bi-Objective Approach. Journal of Systems Science and Systems Engineering. 2018; 27(3): 265-291.

2. Zitzler E, Thiele L, editors. Multiobjective optimization using evolutionary algorithms—a comparative case study. International conference on parallel problem solving from nature; 1998; 1498(6), 292-301.

3. Schott JR. Fault tolerant design using single and multicriteria genetic algorithm optimization: Massachusetts Institute of Technology; 1995.

4. Zitzler E, Deb K, Thiele L. Comparison of Multiobjective Evolutionary Algorithms: Empirical Results. Evolutionary Computation. 2000; 8(2): 173-195.

5. Li JQ, Pan QK, Tasgetiren MF. A discrete artificial bee colony algorithm for the multi-objective flexible job-shop scheduling problem with maintenance activities. Applied Mathematical Modelling. 2014; 38(3): 1111-1132.
